# Long-term trends in colorectal cancer: incidence, localization, and presentation

**DOI:** 10.1186/s12885-020-07582-x

**Published:** 2020-11-10

**Authors:** Øystein Høydahl, Tom-Harald Edna, Athanasios Xanthoulis, Stian Lydersen, Birger Henning Endreseth

**Affiliations:** 1 Department of Surgery, Levanger Hospital, Nord-Trøndelag Hospital Trust, Levanger, Norway; 2 IKOM Department of Clinical and Molecular Medicine, NTNU, Norwegian University of Science and Technology, Trondheim, Norway; 3 Regional Centre for Child and Youth Mental Health and Child Welfare – Central Norway, Faculty of Medicine, Department of Mental Health, Faculty of Medicine, Norwegian University of Science and Technology, Trondheim, Norway; 4 Clinic of Surgery, St Olavs Hospital, Trondheim University Hospital, Trondheim, Norway

**Keywords:** Colorectal cancer, Incidence, Presentation, Trends, Epidemiology

## Abstract

**Background:**

The purpose of this study was to assess trends in incidence and presentation of colorectal cancer (CRC) over a period of 37 years in a stable population in Mid-Norway. Secondarily, we wanted to predict the future burden of CRC in the same catchment area.

**Methods:**

All 2268 patients diagnosed with CRC at Levanger Hospital between 1980 and 2016 were included in this study. We used Poisson regression to calculate the incidence rate ratio (IRR) and analyse factors associated with incidence.

**Results:**

The incidence of CRC increased from 43/100,000 person-years during 1980–1984 to 84/100,000 person-years during 2012–2016. Unadjusted IRR increased by 1.8% per year, corresponding to an overall increase in incidence of 94.5%. Changes in population (ageing and sex distribution) contributed to 28% of this increase, whereas 72% must be attributed to primary preventable factors associated with lifestyle. Compared with the last observational period, we predict a further 40% increase by 2030, and a 70% increase by 2040. Acute colorectal obstruction was associated with tumours in the left flexure and descending colon. Spontaneous colorectal perforation was associated with tumours in the descending colon, caecum, and sigmoid colon. The incidence of obstruction remained stable, while the incidence of perforation decreased throughout the observational period. The proportion of earlier stages at diagnosis increased significantly in recent decades.

**Conclusion:**

CRC incidence increased substantially from 1980 to 2016, mainly due to primary preventable factors. The incidence will continue to increase during the next two decades, mainly due to further ageing of the population.

## Background

Colorectal cancer (CRC) is the fourth most common cancer and the second most common cause of cancer death globally [1]. In 2018 the age-standardized (world) incidence for CRC was 19.7/100,000, higher in males than in females (23.6/100,000 vs. 16.3/100,000) [2]. The distribution of CRC burden varies widely, with increasing incidence in countries where the human development index (HDI) is high [3]*.* Among the Nordic countries, Denmark and Norway have the highest incidence. In Norway the age-standardized (world) incidence of CRC in 2012–16 was 44.9/100,000 in males and 37.4/100,000 in females. The estimated annual increases during the last 10 years were 0.5% among males and 1.1% in females [4]. The incidence of CRC is expected to increase by 33% in 2024–2028, caused mainly by an ageing population [5].

In Western countries CRC is primarily a disease of the elderly, with a peak incidence at around 70 years of age. The aetiology is multifactorial, and most patients are affected in a sporadic manner. Approximately three-quarters have a negative family history [6]. It is well documented that primary preventable causes such as unfavourable diet, obesity, alcohol, smoking, and low physical activity increase the risk of CRC [7].

Based on a continuous exposure to these risk factors, and an expected ageing of the population [8], the number of patients with CRC will grow in the coming years. Knowledge of trends in incidence and clinical characteristics of CRC patients is imperative to tailor diagnostic work-up and treatment, as well as in development of a strategy to meet future changes in the patient population. As the burden on the health care system continues to rise, it will be important to focus on quality and optimal utilization of resources through adequate organization of the services, standardized care pathways, and individualised treatment.

The focus on primary prevention of CRC will continue, but further achievements in reducing CRC incidence are uncertain and will possibly affect future generations. Secondary prevention by screening programs has been proven to reduce the incidence of CRC among attendees in the long run [9]. In Norway, national screening for CRC will be implemented for patients in their mid-fifties in the coming years. Although important, these preventive measures will not have a significant impact on CRC incidence among the rapidly increasing elderly part of the Norwegian population.

This study was designed to analyse epidemiologic trends in patients diagnosed with CRC for nearly four decades, with respect to incidence, presentation of disease, and stage. Secondarily we wanted to use this knowledge to estimate the future burden of CRC.

## Methods

**Fig. 1:**
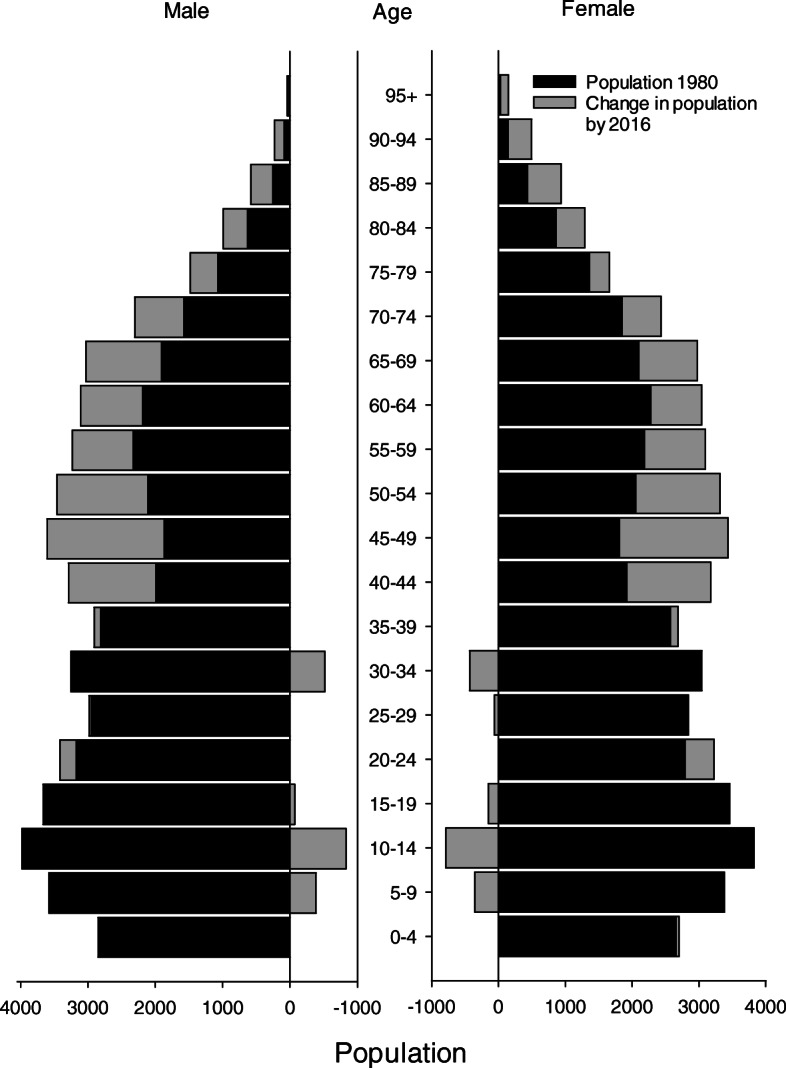
Population in Levanger Hospital’s catchment area in 1980 and in 2016, categorized by age groups

**Fig. 2:**
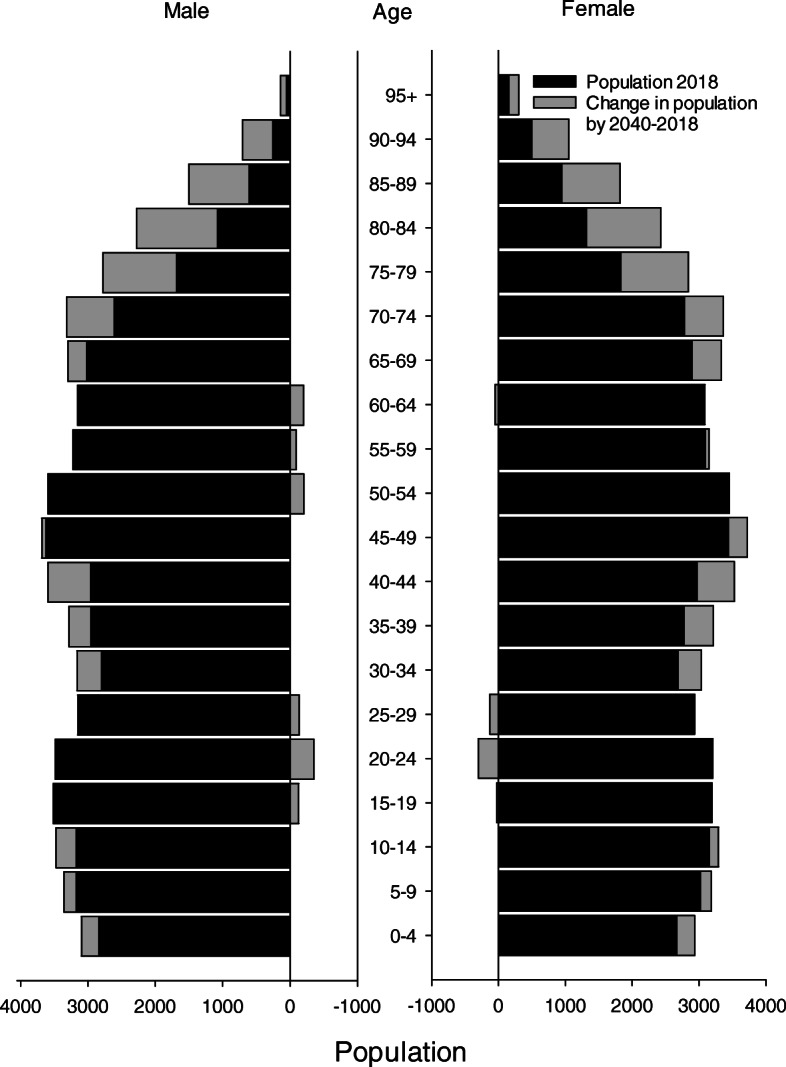
Population in Levanger Hospital’s catchment area in 2018 and estimated population in 2040

All patients with CRC admitted to Levanger Hospital during the 37-year period between January 1980 and December 2016 were included in this study. Levanger Hospital serves as the primary hospital for 10 municipalities in North-Trøndelag County, located in Mid-Norway. The county consists of a long coastline as well as large farmlands and forests. The population lives in small towns, villages, or in rural areas. Agriculture is the most important industry. Mean income and education level are slightly less than the national average. The population rose from 83,890 in 1980 to 99,566 in 2016 (a 19% increase). Figure 1 displays changes in the distribution of age in our catchment area and compares 1980 with 2016. Figure 2 displays the population in 2018 and the estimated population in 2040 [8]. The catchment area remained unchanged throughout the observation period. The patients represented an unselected population.

The patients were identified through the discharge diagnoses in the patient administrative system of the hospital, using ICD-8 diagnosis codes 153.01 to 154.19, ICD-9 codes 153.0 to 154.1, and the ICD-10 codes C18.0 to C20. Patients with cancer of the appendix (C18.1) were excluded. Data were retrieved from the health records of all patients. We registered demographic variables, date of admission, presentation (bowel obstruction or spontaneous perforation), localization of the tumour, and stage according to the *TNM classification of malignant tumours*, 6th edition [10]. The database was confirmed by comparing data from the Norwegian Cancer Registry 1980–2016.

Patients with malignancies other than adenocarcinomas (pseudomyxoma peritonei, neuroendocrine tumours, sarcomas [GIST], and lymphomas) were excluded, leaving 2268 patients with CRC in the final cohort. A histological diagnosis of adenocarcinoma was available in 2159 patients (95.2%). In the remaining 109 patients (4.8%) the diagnosis was made without a biopsy and based upon a combination of CT-findings, CEA level, colon X-ray, clinical findings, and medical history. These were older, frail patients not fit for surgery or oncological treatment.

Colonic cancer located from the caecum to the transverse colon was defined as right sided. Cancer located from the left flexure to the sigmoid colon was defined as left-sided colon cancer [11]. Rectal cancer was defined as cancer located within 15 cm of the anal verge, with upper, middle, and lower rectum distanced 12–15 cm, 6–11 cm, and 0–5 cm from the anal verge, respectively.

We categorized patients into five age groups: < 65 years, 65–74 years, 75–79 years, 80–84 years, and > 85 years. Trends in calendar years were analysed using five-year periods.

### Statistical analysis

The Cochran-Armitage test was used to test for trends in proportions. Logistic regression analysis was used to test for association between intestinal obstruction and perforation at admission as dependent variables and different explanatory variables. Ordinal logistic regression was used to test associations in doubly ordered r x c tables, as in stage by decades. Multinomial logistic regression analysis was used in singly ordered r x c tables, as in the localization of the tumour depending on decade.

The overall incidence of CRC was defined as the number of new cases of CRC in the defined population within 1 year. The incidence rate (IR) was defined as the incidence divided by the total person-time at risk during the same year. The incidence rate ratio (IRR) was defined as the ratio between two incidence rates. The incidence of cancer was analysed using Poisson regression with CRC as the dependent variable and sex, age in five-year intervals (20–24, 25–29, up to 90–94, 95–99), and calendar year from 1980 to 2016 as covariates. Nonlinear relationships were explored by using fractional polynomials [12].

Where relevant, we also adjusted the regression analyses for age, sex, year of diagnosis, and T-stage, which were a priori regarded as plausible confounders.

Age and sex distributions for the 10 municipalities around Levanger Hospital for every year from 1980 to 2016, and information on the expected numbers of males and females by 2030 and 2040, were obtained from Statistics Norway [8].

Two-sided *P*-values < 0.05 were considered significant. Means were reported with the range (minimum to maximum) and standard deviation (SD) where relevant. Ninety-five percent confidence intervals (CI) were reported where relevant. Analyses were carried out in Stata 15, IBM SPSS Statistics 25, and StatXact 9.

## Results

### Study population

**Fig. 3:**
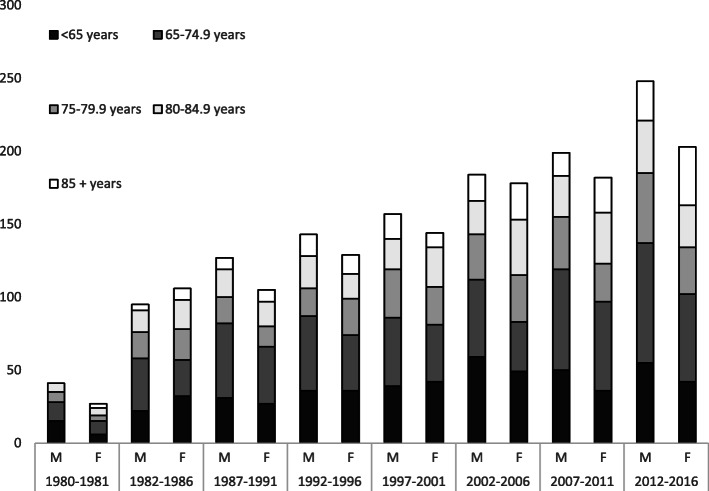
Number of new cases per 5-year period for both sexes and age groups. *The two columns to the very left represent a 2-year period*

The characteristics of the 2268 patients diagnosed with CRC between 1980 and 2016 are presented in Table 1. There were 1194 (53%) males and 1074 females. Two-thirds (*n* = 1551, 68%) of cases were colon cancers. The mean age in colon cancer patients was 72.2 (32.9–96.1, SD 11.1) years in males and 73.1 (20.3–99.6, SD 11.5) years in females. Corresponding numbers for rectal cancer patients were 70.9 (21.6–94.3, SD 10.7) and 70.4 (35.2–97.1, SD 12.0) years, respectively. The mean annual number of new CRC patients from 1980 to 1986 was 38 patients per year compared with 83 patients per year for 2007 to 2016. The group of patients above 85 years increased, representing 6% in the first period and 13% in the last period. We observed non-significant variations in tumour localization throughout the observation period. Figure 3 displays the distribution of patients according to sex and age throughout the study period.

**Table 1:** Characteristics of CRC for each calendar period of admission

Year	1980–1986	1987–1996	1997–2006	2007–2016	Total	*P* value
Patients						0.53 ^a^
Male	136 (51)	270 (54)	341 (51)	447 (54)	1.194	
Female	133 (49)	234 (46)	322 (49)	385 (46)	1.074	
Age						0.004 ^b^
< 65	75 (28)	130 (26)	189 (29)	183 (22)	577	
65–75	83 (31)	179 (36)	173 (26)	272 (33)	707	
75–80	50 (19)	76 (15)	122 (18)	142 (17)	390	
80–85	46 (17)	75 (15)	109 (16)	128 (15)	358	
> 85	15 (6)	44 (9)	70 (11)	107 (13)	236	
Localization						0.29 ^c^
Right colon	99 (37)	177 (35)	252 (38)	327 (39)	855	
Left colon	78 (29)	168 (33)	211 (32)	239 (29)	696	
Rectum	92 (34)	159 (32)	200 (30)	266 (32)	717	
Acute presentation						
Colorectal obstruction	23 (8.6)	57 (11.3)	63 (9.5)	88 (10.6)	231	0.69 ^a^
Perforation	18 (6.7)	17 (3.4)	20 (3.0)	13 (1.6)	68	< 0.001 ^a^
Stage						
I	34 (13)	53 (11)	92 (14)	173 (21)	353	< 0.001 ^d^
II	81 (30)	163 (32)	243 (37)	309 (37)	798	
III	70 (26)	119 (24)	133 (20)	174 (21)	495	
IV	65 (24)	128 (25)	155 (23)	174 (21)	524	
Unknown	19 (7)	41 (8)	40 (6)	2 (0.2)	103	

Values in parenthesis are percentages of column total

^a^ Cochran-Armitage exact trend test

^b^ Ordinal logistic regression with calendar period as covariate

^c^ Multinomial logistic regression with calendar period as covariate

^d^ Ordinal logistic regression with calendar period as covariate, for known stages

### Incidence

The overall unadjusted incidence rate during the 37 years was 66.1/100,000 person-years, 63.1/100,000 person-years in females, and 69.3/100,000 person-years in males. During the first 5 years the overall incidence rate was 43/100,000 person-years, compared with 85/100,000 person-years during the last 5 years.

The incidence rate for CRC increased with every calendar year and with increasing age. The incidence rate increased by 1.2926% for each calendar year when *age and sex* were adjusted for. This corresponded to an increase in 60.8% (1.012926^37^) throughout the entire observation period. When adjusted for *age only*, the increase in incidence rate was 1.2953% per year. Hence, a negligible proportion (0.0027, 1.2953% minus 1.2926%) of the increased incidence rate was attributed to sex. When *neither age nor sex* were adjusted for, the increase in incidence rate was 1.808% for each calendar year, corresponding a total increase of 94.1% (1.01808^37^). The increase in incidence rate attributed to the ageing of the population and sex distribution was 0.512% (1.808% minus 1.2926%), equivalent to a 28% relative increase (0.512/1.808 = 28%). Factors *other than* sex *and ageing of the population* were the main reasons for the incidence increase, and 72% of the observed increase must be attributed to them.

Table 2 shows the IRRs of CRC as a function of age and calendar year, for males and females separately. There was a significant increase in incidence rate for both sexes with calendar year and age, apart from left-sided colonic cancer for women and rectal cancer for men.

**Table 2:** Factors associated with CRC. Adjusted IRRs from Poisson regression. Calendar year and age as covariates

	Male		Female	
	IRR (CI)	*P* value	IRR (CI)	*P* value
Total colorectal cancer *n* = 2173 ^a^				
Calendar year	1.0133 (1.0078–1.0189)	< 0.001	1.0127 (1.0068–1.0186)	< 0.001
Age (per 5 years)	1.0807 (1.0764–1.0850)	< 0.001	1.0691 (1.0650–1.0732)	< 0.001
Right sided colonic cancer *n* = 841				
Calendar year	1.0208 (1.0111–1.0306)	< 0.001	1.0148 (1.0059–1.0238)	0.001
Age (per 5 years)	1.0887 (1.0811–1.0964)	< 0.001	1.0798 (1.0730–1.0866)	< 0.001
Left sided colonic cancer *n* = 686				
Calendar year	1.0155 (1.0055–1.0256)	0.002	1.0093 (0.9990–1.0197)	0.077
Age (per 5 years)	1.0797 (1.0721–1.0872)	< 0.001	1.0627 (1.0557–1.0697)	< 0.001
Rectal cancer *n* = 646				
Calendar year	1.0042 (0.9950–1.0136)	0.37	1.0130 (1.0013–1.0249)	0.030
Age (per 5 years)	1.0743 (1.0674–1.0813)	< 0.001	1.0607 (1.0528–1.06856)	< 0.001

^a^ Ninety-five patients admitted to Levanger Hospital from the area of Namsos Hospital, mostly because of centralization of rectal cancer during the later years, have been excluded from these incidence analyses. They were not included because that area was not an original part of the primary population area of Levanger Hospital

**Fig. 4:**
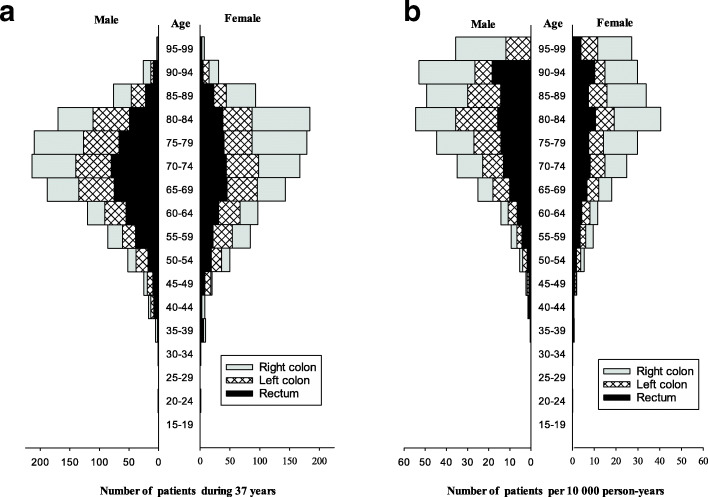
**a** Number of new cases with colorectal cancer during a 37-year period (left). **b** Number of new cases per 10,000 person-years (right)

Figure 4a shows the absolute number of patients distributed by 5-year age-groups and sex. Figure 4b shows the same patients compared with the number of persons of the same sex and age in this area of Trøndelag. The figure shows that CRC was becoming more frequent as age increased.

**Fig. 5:**
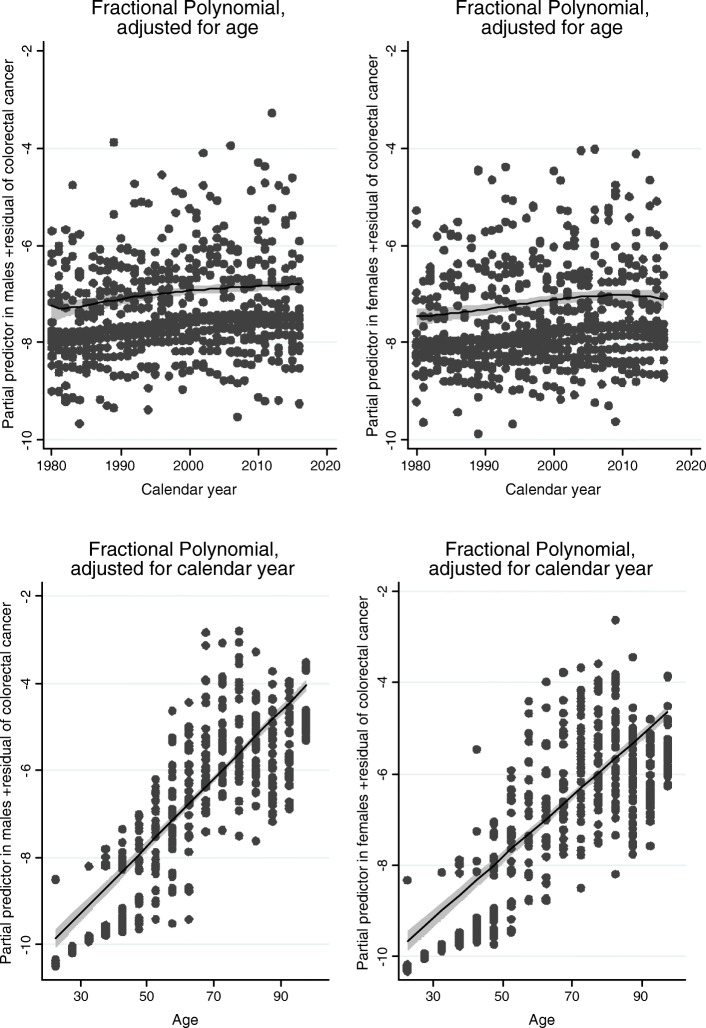
Effects of calendar year and age on the number of patients presenting with colorectal cancer. Effects of calendar year and age on the number of patients presenting with colorectal cancer in a Poisson regression with fractional polynomials (confidence intervals are shaded grey, logarithmic scale on the y-axis, *males in the figures to the left*). The increase for each calendar year diminished in the later years. The effect of age was linear in both males and females

Figure 5 shows the results of possibly nonlinear effects of age and calendar year for CRC, using fractional polynomials. The lower figures in Fig. 5 show a straight line as a function of age, for both males and females. This confirms that the assumption of a linear effect of age on the logarithm of incidence is a good approximation to reality in our data. In other words, the risk of colorectal cancer increases by a factor of approximately 1.081 per 5 years for males and 1.069 per 5 years for females (Table 2) throughout the lifetimes we have in our study. Regarding the effect of calendar year, the upper two figures indicate a nonlinear effect of calendar year: the increase in incidence was largest in the first years from 1980, and seems to have flattened out between 2000 and 2010. From around 2000 there was less of an increase or no increase in age-adjusted incidence.

### Predicting the future burden of colorectal cancer

**Fig. 6:**
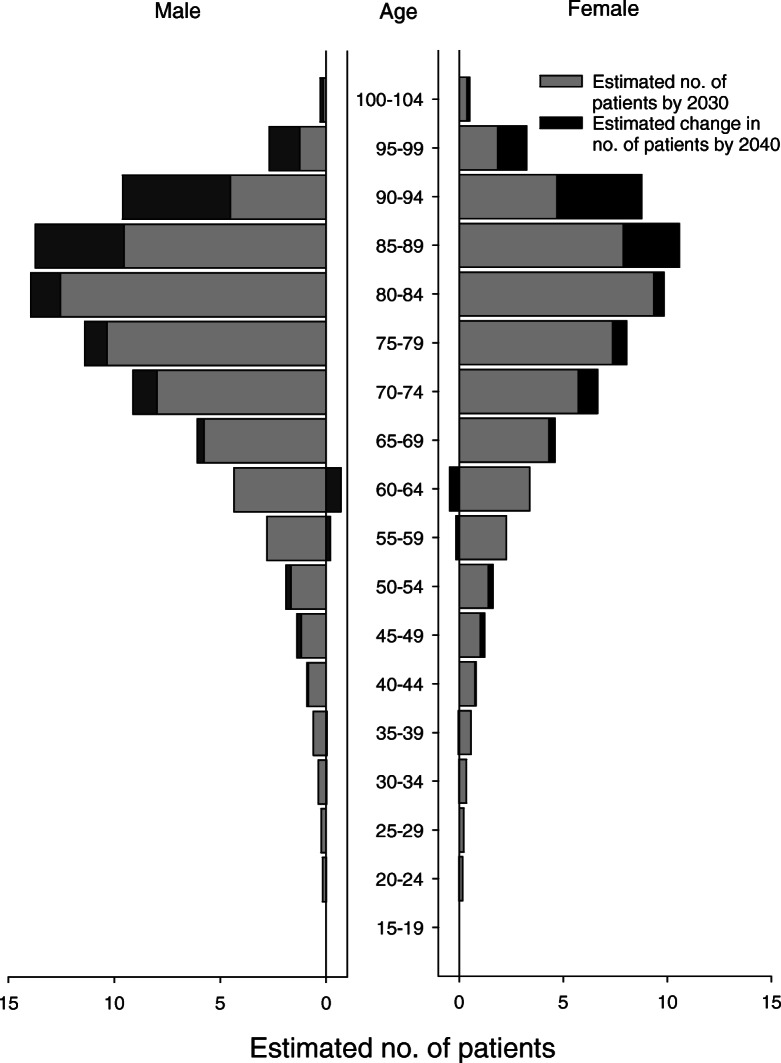
Estimated numbers of new cases with CRC by 2030 and 2040

The results of the Poisson analysis with fractional polynomials showed that the calendar-year effect seemed to flatten out around 2000 to 2010. The predicted numbers of CRC cases in future years are based on the mean incidence rates for the latest 10 years of the study period (2007–2016) for each 5-year age group, separately for males and females. A Poisson model was used to predict the number of cases occurring by 2030 and by 2040; see the results in Fig. 6. In the year 2030, the model estimates a total of 116 (50% prediction interval: 109–124) new CRC patients in our catchment area, including 65 males and 52 females. Corresponding numbers for the year 2040 are 79 males and 62 females, totalling 141 patients (50% prediction interval: 133–150).

### Stage

Stage for each time period is shown in Table 1. The proportion of earlier stages increased significantly in recent decades. There were substantially fewer patients with unknown stage. Table 3 shows stage as a dependent variable with regard to sex, age, decade, and localization of the obstructing tumour. The results of multivariable analyses showed that older age, diagnosis in recent years, and distal location were associated with earlier stages.

**Table 3:** Stage at presentation. Ordinal logistic regression with known stage at presentation as the dependent variable.^a^

	Unadjusted odds ratio	*P* value	Adjusted odds ratio	*P* value
Female sex	1.03 (0.89–1.20)	0.69	0.99 (0.84–1.15)	0.85
Age	0.994 (0.987–1.001)	0.080	0.99 (0.986–0.999)	0.046
Year of diagnosis	0.981 (0.974–0.989)	< 0.001	0.98 (0.974–0.989)	< 0.001
Location				
Right colon	1		1	
Left colon	0.86 (0.72–1.03)	0.11	0.83 (0.69–1.004)	0.055
Rectum	0.64 (0.53–0.77)	0.004	0.62 (0.51–0.75)	< 0.001

^a^ Sex, age, year of diagnosis, and location of the primary tumour as covariates. Unadjusted, and adjusted for age, sex, and year

### Colorectal obstruction and perforation

Acute colorectal obstruction was the presenting symptom in 231 of 2268 patients (10.2%). Table 4 shows presentation with acute colorectal obstruction with regard to sex, age, calendar year, and localization of the obstructing tumour. Multivariable analysis showed that acute colorectal obstruction was associated most commonly with tumours in the left flexure and the descending and sigmoid colon. It was significantly less frequent with rectal tumours. There were no associations between colorectal obstruction and sex or age.

**Table 4:** Colorectal obstruction. Logistic regression with colorectal obstruction at presentation as the dependent variable^a^

	Colorectal obstructions (%)	Unadjusted odds ratio	*P* value	Adjusted odds ratio	*P* value
Female sex	121/1074 (11.3)	1.25 (0.95–1.64)	0.11	1.18 (0.88–1.59)	0.28
Age		1.011 (0.999–1.024)	0.08	1.01 (0.996–1.023)	0.18
Year of diagnosis		1.004 (0.991–1.017)	0.57	1.02 (1.001–1.031)	0.037
T-Stage			< 0.001		< 0.001
1–2	3/418 (0.7)	1		1	
3	134/1202 (11.1)	17.4 (5.50–55)	< 0.001	15.6 ((4.90–49)	< 0.001
4	71/437 (16.2)	26.8 (8.4–86)	< 0.001	29.7 (9.16–96)	< 0.001
Unknown	11/89 (12.4)	19.5 (5.3–72)	< 0.001	20.3 (5.40–76)	< 0.001
Location			< 0.001		< 0.001
Caecum	31/288 (10.8)	7.80 (2.35–26)	0.001	6.40 (1.90–22)	0.003
Ascending colon	26/310 (8.4)	5.92 (1.77–20)	0.004	4.98 (1.47–16.9)	0.010
Right flexure	8/99 (8.1)	5.69 (1.47–22)	0.012	4.64 (1.19–18.1)	0.027
Transverse colon	22/158 (13.9)	10.5 (3.07–36)	< 0.001	9.39 (2.72–32)	< 0.001
Left flexure	21/62 (33.9)	33.1 (9.44–116)	< 0.001	27.5 (7.61–99)	< 0.001
Descending colon	19/93 (20.4)	16.6 (4.77–58)	< 0.001	18.7 (5.24–66)	< 0.001
Sigmoid	83/541 (15.3)	11.7 (3.66–38)	< 0.001	11.8 (3.63–38)	< 0.001
Proximal rectum	11/220 (5.0)	3.40 (0.94–12.4)	0.063	2.72 (0.70–10.5)	0.15
Middle rectum	7/300 (2.3)	1.55 (0.40–6.05)	0.53	1.69 (0.43–6.70)	0.45
Distal rectum	3/197 (1.5)	1		1	

^a^ Sex, age, year of diagnosis, and location of the primary tumour as covariates. Unadjusted, and adjusted for age, sex, year of diagnosis, and T-stage

Spontaneous colorectal perforation occurred in 68 of 2268 patients (3.0%). Table 5 shows spontaneous colorectal perforation with regard to sex, age, calendar year, and localization of the obstructing tumour. Perforation was associated with tumours in the descending colon (5.4%), caecum (4.9%), and sigmoid colon (4.8%). Perforation became significantly less frequent as time passed, and was not associated with sex or increasing age. In the last period perforation occurred in 1.6% of the patients.

**Table 5:** Spontaneous colorectal perforation. Logistic regression with spontaneous colorectal perforation at presentation as dependent variable^a^

	Perforations (%)	Unadjusted odds ratio	*P* value	Adjusted odds ratio	*P* value
Female sex	30/1074 (2.8)	0.87 (0.54–1.42)	0.59	0.81 (0.48–1.35)	0.42
Age		0.99 (0.97–1.005)	0.13	0.98 (0.96–1.01)	0.14
Year of diagnosis		0.96 (0.93–0.98)	< 0.001	0.97 (0.94–0.99)	0.009
T-Stage			< 0.001		< 0.001
1–2	1/418 (0.2)	1		1	
3	27/1202 (2.2)	9.58 (1.30–71)	0.027	8.57 (1.16–64)	0.036
4	38/437 (8.7)	39.7 (5.43–291)	< 0.001	36.7 (4.97–272)	< 0.001
Unknown	0/89 (0)	0	0.997	0	0.997
Location					
Caecum	14/288 (4.9)	10.0 (1.31–77)	0.027	9.25 (1.18–73)	0.034
Ascending colon	5/310 (1.6)	3.12 (0.37–28)	0.03	3.59 (0.41–31)	0.25
Right flexure	3/99 (3.0)	6.13 (0.63–60)	0.12	5.17 (0.52–52)	0.16
Transverse colon	2/158 (1.3)	2.51 (0.23–28)	0.45	2.38 (0.21–27)	0.49
Left flexure	2/62 (3.2)	6.53 (0.58–73)	0.13	5,60 (0.49–64)	0.17
Descending colon	5/93 (5.4)	11.14 (1.28–97)	0.029	13,7 (1.54–123)	0.019
Sigmoid	26/541 (4.8)	9.90 (1.33–73)	0.025	11.9 (1.57–90)	0.017
Proximal rectum	5/220 (2.3)	4.56 (0.53–39)	0.14	5.40 (0.61–48)	0.13
Middle rectum	5/300 (1.7)	3.32 (0.39–29)	0.28	2.68 (0.29–25)	0.38
Distal rectum	1/197 (0.5)	1		1	

^a^ Sex, age, year of diagnosis, and location of the primary tumour as covariates. Unadjusted, and adjusted for age, sex, year of diagnosis, and T-stage. Distal rectal cancer was used as the reference location

## Discussion

### Incidence

This observational survey was completed to assess epidemiological and clinical trends in CRC over a 37-year period, and to estimate future changes in the patient population. The overall incidence rate of CRC increased by 90% during the study period. Of this observed increase, 28% was attributed to changes in the population (age and sex), whereas 72% was related to other factors. According to our estimates, the number of new CRC patients, particularly octogenarians, will continue to rise in the coming years. We shall expect a 40% increase in 2030 and a 70% increase in 2040, compared with mean incidence rates the past 10 years.

The local incidence rate in our catchment area was somewhat below the national level in 1980–1984, but increased to the national level during the last 5 year period of the study [13]. Our county, as well as other rural areas of Norway, has undergone some urbanisation throughout this period. Differences in lifestyle among Norwegian citizens living in the cities and in the countryside are diminishing, and the population is to an increasing extent exposed to the same risk factors. Global patterns show a marked increase in the incidence of CRC in countries adopting modern Western living habits [3]. Norway has enjoyed rapid social and economic development since the 1970s, in great extent due to the oil industry. There has been an increase in the rates of obesity and diabetes in our county [14, 15], as well as in the rest of the country. Only 30% of the Norwegian population fulfil the recommended level of daily physical activity. On the other hand, there has been a decrease in daily smokers, from 36% in 1980 to 12% in 2018 [8].

Other reports have findings comparable to ours, attributing a large proportion of the increase in CRC incidence to preventable risk factors [16]. In the United Kingdom, one-third of all cancers are attributed to smoking, and one third to diet, nutrition, and physical activity [17]. Despite public initiatives to reduce the exposure to known risk factors – for example, advice regarding physical activity, smoking and diet – incidence levels have increased. From the present report, it seems that the effect of preventable risk factors on the incidence of CRC reached a peak around 2000–2010, with a more stable incidence in later years. Whether this is an effect of increased knowledge of risk factors and consequent behavioural changes in the population or indicates a maximum steady-state level of exposure to these risk factors in the population is disputable.

CRC is a disease with a multifactorial genesis primarily affecting the population in a sporadic manner, with a peak incidence in persons older than 70 years of age. The proportion of elderly patients has increased throughout our observation period, and this trend will continue in the future. Especially noticeable is the increasing number of patients above 85 years of age. According to the Norwegian national guidelines on CRC, a 33% increase in incidence is expected by 2024–2028, mainly due to ageing of the population [5]. Our predictions coincide with the numbers presented in those guidelines.

Among the OECD countries, Norway is fourth in life expectancy. Other countries at the top of this list are also high HDI countries with high incidences of CRC (e.g., Switzerland, Japan, Australia, and Sweden) [18]. Norwegian life expectancy has increased by 7.5 years since the 1980s, and we found that 28% of the increased incidence in CRC could be attributed to increased age.

The Norwegian health care system is fully funded by the government. Hence, every Norwegian citizen has access to state-of-the-art medical services, and can seek medical help at any time, regardless of income. Colonoscopy and CT are nowadays, in contrast with the 1980s, considered low-threshold examinations. General practitioners can refer patients for these examinations within 9 calendar days (fast-track examination), if cancer is suspected. This may contribute to the high incidence levels, earlier stages detected, and decrease in the number of perforations at presentation observed in Norway recently.

Decreasing incidences of CRC are observed in countries with established screening programs [19, 20]. A national Norwegian screening program is currently being planned, enrolling patients at the age of 55 years. An increase in incidence rates must be expected before the incidence rates decline. Implementation of this screening program will not affect incidence among patients aged above 55 years at the time of implementation. During the first years after the Second World War, Norway experienced all-time-high birth rates. As life expectancy continues to increase in Norway, these large cohorts of elderly citizens not undergoing screening will result in an increased number of elderly CRC patients. In combination, these two factors will contribute to a peak in CRC incidence in the coming years. In a longer time-frame, however, we might observe falling incidence rates as the result of screening. Declining birth rates in Norway may augment this change in an even longer perspective.

### Stage

In this study there was a trend towards earlier stages at diagnosis in recent decades. This might reflect more awareness of the disease among both patients and primary care physicians, better access to colonoscopy, and a more widespread use of CT with improved quality. These findings are contrary to other studies, which have reported unchanged or increasing rates of advanced stages with time [21–23]. Screening-detected cancer patients present with earlier stages of disease compared with non-screening-detected patients [24–28]. The patients in this study were all diagnosed before the introduction of systematic screening for CRC, indicating that the shift towards earlier stages at presentation will continue in the future. Distal localizations had earlier stages compared with proximal tumours, in accord with previous reports [29, 30].

### Colorectal obstruction and perforation

Previous reports found emergency presentation of colorectal cancer in 9–32% of the patients, primarily due to colorectal obstruction and bowel perforation [31–37]. The incidence of complete obstruction has been reported as 8.3 to 22.9%, and the perforation rates from 2.3 to 3.6% [31, 34, 36–42]. We found comparable rates, of 10.5 and 3.1% of the patients, respectively. Neither colorectal obstruction nor spontaneous perforation was associated with age in the present study, contrary to findings in previous reports [42]. Primary tumour localization to the left flexure had the highest rate of obstruction, at 34%. Two other studies found that almost half of the tumours with this localization resulted in obstruction [42, 43]. The rate of spontaneous perforation diminished significantly during the study period. This might be due to a more effective health care system with shorter waiting times prior to surgery in patients presenting with obstructive symptoms or stenotic tumours at the time of colonoscopy.

### Strengths and weaknesses

This study included a complete cohort of patients diagnosed with CRC over 37 years at a single institution serving a catchment area that remained unchanged throughout the study period. All patients with suspected CRC in our region were referred to our hospital for diagnostic work-up. Data were accessible at an individual level, and completed with data from the Norwegian Cancer Registry. Preoperative examinations, treatment and follow-up followed local guidelines (standardized policies) throughout the period, and similar guidelines were implemented at a national level in 2009. As all patients were included, we avoided selection bias. The population in our county is a stable population, suitable for epidemiologic studies [44]. The study reflects the epidemiology of elective as well as emergency admission of patients with colorectal cancer on a population basis.

The retrospective design implies certain weaknesses. The quality of the database was dependent on the quality of the individual records of the patients. By combining the data from the Norwegian cancer registry with our own database, we believe that the data used to calculate incidences were nearly complete. We may have missed some old, frail patients with symptoms of CRC who were treated at home or in nursing homes, without further investigation. The incidence in very old persons might thus be higher than reported.

Predictions of future cancer incidence depend upon a number of uncertain factors, and numbers must be interpreted with caution [45]. The numbers of CRC cases predicted to occur by 2030 and by 2040 in the present study assumed the same age- and gender-specific incidence rates as the means of the rates that were observed during 2007–2016.

### Future perspectives

The most striking results of predicting future CRC cases occurring by 2030 and by 2040 were the continuous increase in CRC cases in our catchment area and the high numbers of octogenarians, the latter reflecting the impact of increased life expectancy in Norway in the coming years. Awareness of risk factors and systematic screening may reduce the incidence rates. Measures to also reduce the risk of CRC in the elderly non-screened parts of the population should be considered.

In the coming years, the Norwegian health care system must prepare for an increasing number of patients diagnosed with CRC. A large proportion of these patients will be 80–90 years of age. The planned national screening program will not have an impact on CRC incidence among inhabitants aged above 55 years. In the screened part of the population, an initial increase in incidence and a shift towards earlier stages of CRC at presentation should be expected. In the long run, both screening and changes in the population may result in a decline in CRC incidence. Knowledge of these changes in patient volume and characteristics is imperative in order to establish a rational and effective organization of health services to accommodate these patients.

The current study demonstrates that a substantial number of cancer cases can be attributed to preventable causes. Increased knowledge concerning these causes is imperative to complete the puzzle regarding risk factors and disease development. The adverse development regarding obesity and lifestyle-related diseases accentuates the reality that current primary preventive strategies lack effectivity. Given the fact that more than two-thirds of CRC cases might be preventable, a key question is whether changes in these factors can be expected, and what impact this might have on disease development.

## Conclusion

The CRC incidence rate increased by 90% from 1980 to 2016, mainly due to preventable factors. The incidence will continue to increase during the next two decades, primarily because of further ageing of the population. Continuous focus on preventive strategies, as well as awareness of changes in patient characteristics and volume are imperative to ensure adequate capacity, high quality and efficient patient care in the future.

## Data Availability

The dataset used for this study is located on a secure server in the Hospital’s data system. Requests regarding the dataset can be addressed to Øystein Høydahl. The database was confirmed by comparing data from the Norwegian Cancer Registry 1980–2016. Data obtained from the Norwegian Cancer Registry is available by application to the registry.

## Abbreviations

CI: Confidence interval
CRC: Colorectal cancer
HDI: Human development index
IR: Incidence rate
IRR: Incidence rate ratio
SD: Standard deviation
